# Strategies adopted to manage physical and psychosocial challenges after returning home among people with stroke: A qualitative study

**DOI:** 10.1097/MD.0000000000025026

**Published:** 2021-03-12

**Authors:** Suzanne Hoi Shan Lo, Janita Pak Chun Chau, Anne Marie Chang

**Affiliations:** a The Nethersole School of Nursing, Faculty of Medicine, The Chinese University of Hong Kong, Hong Kong; b School of Nursing, Faculty of Health, Queensland University of Technology, Brisbane, Queensland, Australia.

**Keywords:** health services needs and demand, qualitative research, self-management, stroke, stroke rehabilitation

## Abstract

Stroke survivors encounter various physical and psychosocial challenges after hospital discharge. Systematic reviews consistently suggest the importance of self-management in promoting post-stroke recovery. However, stroke survivors’ performance of self-management behaviors after returning home is poorly understood. This study was conducted to explore how stroke survivors manage their life after returning home from the hospital. This was a qualitative study with individual, semi-structured interviews. We recruited a purposive sample of adults who had a first or recurrent ischemic or hemorrhagic stroke and currently lived at home. Participants were asked about their post-stroke experiences, challenges encountered, and strategies adopted for managing post-stroke conditions. Data were transcribed verbatim and analyzed using thematic analysis. A total of 30 stroke survivors (mean age = 61.97 years, SD = 10.20) were interviewed. Most were men (n = 18), married (n = 25), and retired (n = 21). Two-thirds had experienced an ischemic stroke. Five key themes emerged: pursuing lifelong learning to live well after a stroke; reinterpreting unpleasant experiences as new learning opportunities; engaging in life activities to better adapt to post-stroke challenges; being confident in oneself to persevere in self-management behaviors; and continuing to accept the current self and explore the new self. Participants regarded learning as a prerequisite for improving their affected functions and managing uncertainties in recovery. Learning requires self-participation, building self-efficacy and positive outcome expectations, testing and adapting strategies to one's own health conditions, and engaging in leisure or social activities. These findings will guide future development of interventions for enhancing stroke survivors’ recovery outcomes.

## 1 Introduction

Stroke has remained the second leading global cause of death since 2000 and has been ranked the fourth leading cause of death in Hong Kong since 2003.^[1,2]^ Stroke prevalence is estimated to increase as the world's aging population increases.^[1,3]^ Stroke is an important cause of disability. Even survivors of mild stroke experience chronic symptoms such as limb weakness, decreased concentration, and memory loss. Some stroke survivors may experience frustration, anxiety, or even depressive moods consequential to uncertainties regarding rehabilitation or recurrent stroke. Hardships in regaining optimal independence after stroke are consistently reported.^[4–6]^ In addition, aging or other comorbid conditions such as diabetes can further complicate these post-stroke physical and psychosocial challenges, which significantly decrease survivors’ health-related quality of life reported >2 years post-stroke.^[7,8]^

Post-stroke recovery often extends far beyond the patient's hospital discharge. Stroke survivors often require long-term and persistent support to address their diversified daily challenges after returning home.^[9]^ Studies have reported that survivors may rely on their healthcare providers or caregivers to manage their health and may doubt their own likelihood of improvement. Consequently, this may hinder their motivation and engagement in continued rehabilitation.^[9]^ Therefore, international evidence-based clinical guidelines recommend ongoing self-management support to stroke survivors to improve their health outcomes and use of healthcare services after discharge from hospitals.^[10,11]^ Stroke self-management refers to one's capability to manage stroke-related changes, including symptoms, treatment, and physical and psychosocial challenges. Effective self-management requires stroke survivors to learn core self-management skills, including setting goals, planning actions, solving problems, making decisions, communicating with healthcare providers, and using community resources. Underpinning these skills is survivors’ acceptance of responsibility for their self-management behaviors, such as exercising and managing psychological distress.^[12]^

A recent meta-review of 13 systematic reviews reported that stroke self-management interventions were associated with significantly improved daily living activities, reduced dependence, and fewer deaths.^[13]^ Another systematic review of three randomized controlled trials found that Bandura construct of self-efficacy is the most common theoretical framework underpinning self-management interventions. These theory-based interventions showed promise in enhancing stroke survivors’ self-efficacy and health-related quality of life.^[14]^ Self-efficacy is one's judgment of their ability to perform a behavior. Outcome expectation, a construct closely related to self-efficacy, refers to one's belief in the likelihood of outcomes after performing a behavior.^[15]^ Systematic reviews have consistently reported this construct to be significantly positively associated with stroke survivors’ self-efficacy and health-related quality of life.^[15,16]^ However, survivors’ self-management of post-stroke challenges after returning home, and their perceived importance of self-efficacy and outcome expectations in promoting recovery after stroke remains poorly understood.

## 2 Aims

This study was conducted to explore how stroke survivors manage their lives after returning home from the hospital, including adaptations and adjustments made to address post-stroke physical and psychosocial challenges, strategies adopted to facilitate participation in self-management behaviors, perceived importance of self-efficacy and outcome expectations in promoting their recovery, and health needs in performing self-management behaviors.

## 3 Methods

### 3.1 Study design

This was a qualitative study using individual, semi-structured, in-depth interviews with stroke survivors.

### 3.2 Participants and setting

Participants were recruited from 2 stroke support groups and a community rehabilitation organization offering rehabilitation and social care services for people with chronic conditions, including stroke, in Hong Kong. A convenience sample of participants were recruited if they were/had: aged 18 years or older, clinically diagnosed with a first or recurrent ischemic or hemorrhagic stroke,^[17]^ been discharged from a hospital to home, able to communicate in Cantonese, willing to share their experiences of managing their physical and/or psychosocial challenges after stroke, and a Montreal Cognitive Assessment score below the second percentile.^[18]^ Survivors were excluded if they resided in residential care homes, had been diagnosed with a mental condition, had severe dysphasia, or had hearing problems that could not be corrected by hearing aids.

### 3.3 Ethical consideration

The Joint Chinese University of Hong Kong-New Territories East Cluster Clinical Research Ethics Committee, Hong Kong (Ref. No.: 2014.375-T, 2018.009) and the Human Research Ethics Committee of the Queensland University of Technology, Australia (Ref. No.: 1400000333) approved the study. All participants provided written informed consent before data collection, after which they received a card indicating their involvement in the study and a means of urgent contact. Data collected via interviews and questionnaires were anonymized and used for research purposes only. All information collected was kept strictly confidential. The federal and institutional ethical standards, Declaration of Helsinki, International Conference on Harmonization-Good Clinical Practice and Hong Kong Personal Data (Privacy) Ordinance were adhered to throughout the study.

### 3.4 Data collection

The research team developed a semi-structured interview guide. The first author (SHSL) individually interviewed eligible participants in a private room at a university or a community rehabilitation center. Participants were invited to share their experiences about post-stroke recovery, physical and psychosocial challenges encountered upon returning home, how they developed their own adaptation or adjustment methods, strategies that they perceived as effective to facilitate participation in self-management behaviors, perceived importance of enhancing self-efficacy and positive outcome expectations, and needs for self-management support. All interviews were conducted in Cantonese, lasted for approximately 90 minutes, and were audio-recorded and duplicated to avoid accidental data loss. Additionally, each participant was asked to complete a demographic and clinical information questionnaire, including age, sex, marital status, educational level, mobility and functional status, history of stroke, and past and current medical health and social histories. The interviewer also recorded field observations of the participants’ self-management behavioral performance and their gestures or facial expressions during interviews.

### 3.5 Data analysis

An independent research assistant transcribed the interview data verbatim from the audio recordings. The 6 phases of thematic analysis outlined in Braun and Clarke^[19]^ were applied for data analysis. Two independent researchers (SHSL and JPCC) read the typed transcripts and developed initial codes based on the study aims, grouping them under themes and subthemes. The theme names were carefully considered to ensure that the concepts being described were best represented. Written notes of field observations during the interviews were also compared with the interview data to corroborate study findings.

## 4 Results

### 4.1 Participants

Thirty stroke survivors (mean age = 61.97 years, SD = 10.20) were recruited and individually interviewed. Most were men (n = 18), married (n = 25), and retired (n = 21). Twenty had experienced an ischemic stroke, and 25 had experienced a first-ever stroke (mean duration after first stroke = 10.93 years, standard deviation = 7.20). Most survivors walked with a stick (n = 19), and 10 had dysphasia, expressing themselves at a slower pace. Table 1 summarizes the participants’ demographic and clinical information. Five themes were derived from the interview data.

**Table 1:** Demographic and clinical characteristics of the participants (n = 30).

ID No.	Age	Gender	Marital status	Educational level	Employment	First/recurrent stroke	Duration since first stroke, yrs	Type of stroke	Mobility level
1	64	M	Married	Primary	Retired	First	25	I	Stick
2	60	M	Married	Primary	Retired	First	16	I	Unaided
3	60	F	Married	Primary	Retired	Recurrent	19	H	Stick
4	52	M	Married	Primary	Full-time	Recurrent	6	I	Unaided
5	65	M	Married	Primary	Retired	First	6	I	Stick
6	70	M	Married	Illiterate	Retired	First	5.5	I	Stick
7	57	F	Single	Secondary	Part-time	First	25	I	Stick
8	54	M	Married	Primary	Retired	First	5	H	Stick
9	66	M	Single	Secondary	Retired	First	14	I	Stick
10	35	M	Single	Secondary	Retired	First	6	H	Stick
11	64	F	Single	Primary	Retired	First	11	I	Stick
12	56	F	Married	Secondary	Retired	Recurrent	24	H	Unaided
13	70	F	Married	Primary	Housewife	First	17	H	Unaided
14	80	F	Married	Secondary	Retired	Recurrent	7	I	Stick
15	60	F	Married	Primary	Housewife	First	14	I	Stick
16	76	M	Married	Secondary	Retired	First	2	I	Stick
17	70	F	Married	Primary	Retired	Recurrent	5	I	Stick
18	73	F	Married	Tertiary	Retired	First	7	I	Unaided
19	36	M	Married	Tertiary	Employed	First	3	H	Unaided
20	50	M	Married	Secondary	Employed	First	4	I	Stick
21	67	M	Married	Secondary	Retired	First	11	H	Unaided
22	52	M	Married	Secondary	Unemployed	First	10	H	Unaided
23	71	M	Married	Secondary	Retired	First	3.4	H	Stick
24	62	M	Married	Secondary	Retired	First	3.66	I	Stick
25	69	M	Married	Primary	Retired	First	12	I	Unaided
26	61	F	Single	Secondary	Unemployed	First	20	I	Stick
27	69	F	Married	Primary	Retired	First	10	I	Stick
28	62	M	Married	Secondary	Retired	First	14	I	Unaided
29	69	M	Married	Secondary	Retired	First	3.75	I	Stick
30	59	F	Married	Secondary	Unemployed	First	3.6	H	Unaided

F = female, H = hemorrhagic, I = ischemic, M = male.

### 4.2 Theme 1: Pursuing lifelong learning to live well with stroke

All participants regardless of stroke severity consistently described their recovery as chronic, dynamic, and complex. They encountered diversified health challenges after returning home. It ranged from basic self-care activities such as pouring water into a cup to drink or grasping a towel to wash their face, to dealing with cyclical frustrating emotions such as worry and uncertainty, grieving for loss of independence or past achievements, or altered social relationships (e.g., shifting from caregiving to receiving care, or inadequate understanding by others due to their invisible symptoms such as memory loss or reduced concentration). None expressed an endpoint of full recovery. To live well with these interrelated and changing physical and psychosocial challenges, participants mentioned that they had to constantly learn new knowledge and skills:

“I used to joke with my friends that I had become like a kindergarten student, learning from the basics … how to walk, groom, and even eat, go to the toilet … In life, we keep learning. It's just that I am learning to do everything again, it's no different from learning itself.” (Participant 21, man, age 67)

“As long as the activities or information are helpful in improving my [affected] arm and leg, I will definitely learn them.” (Participant 14, woman, age 70)

The knowledge and skills learned differed among participants, depending on their health needs and recovery stage. They prioritized physical needs more in the first year post-stroke, then became more concerned with social needs and reintegration into the community in the following years. However, they shared a consistent goal of learning to adapt to post-stroke challenges and ultimately to lead an optimally independent life:

“I have been getting along well with my ‘old friend,’ [the paralyzed arm] … I will not and cannot get rid of it. I developed my own way of drying clothes using my unaffected arm … It takes nearly triple the time, but I can still do it.” (Participant 18, woman, age 73)

“I used to be surprised when my [affected] leg was better on some days and worse on others … Now, I know this pattern … When the worse days come, I rest more at home … holding my anger … not like the past me.” (Participant 28, man, age 62)

While most participants acknowledged the importance of families and caregivers in supporting their recovery, all participants affirmed the importance of their own self in contributing to successful learning during their recovery:

“My health is in my own hands. I am the only person who can take full responsibility of my health.” (Participant 02, man, age 65)

“Your wife, husband, son, daughters may take you to the doctor, but they cannot help you move your [affected] legs to gain strength. You must move your legs, and only you know how to move in a way that is most comfortable or helpful.” (Participant 12, woman, age 60)

### 4.3 Theme 2: Reinterpreting unpleasant experiences as new learning opportunities

When asked how they developed strategies to facilitate participation in self-management behaviors, participants shared abundant stories about unpleasant experiences, ranging from multiple failures despite tremendous hard work to negative attitudes or rude behaviors from others. They commonly described themselves as initially defeated by these experiences, but later realized that they did not want to continue to behave like that. They then determined to transform these unpleasant experiences into new learning opportunities and drives for further improvement:

“At that time after returning home, I was unable to sit steadily. I had no idea about what I could do to help me out at the beginning. I just felt extremely sad and embarrassed when my sister-in-law helped me shower. I hate this … then I knew the first thing to do was to train my sitting balance … that's how my strategy started, I think.” (Participant 07, woman, age 57)

A participant indicated that he improved his arm function after “harsh” training by his wife:

“I was initially angry with my wife for not helping me put on clothes … I was forced to move my body to get dressed. I sweat a lot and would wet the clothes. Gradually, I found it helpful in training my upper limb function and I no longer required her help.” (Participant 05, man, age 60)

Another participant said she was determined to work hard to regain independence after one specific negative experience:

“One day my husband and son were out for a haircut … I was thirsty, so I tried to boil water. My right [affected] arm was too weak to hold the kettle and it fell onto the ground. The hot water nearly splashed on my legs. I cried. I felt sorry about how I couldn’t even boil water for myself.” (Participant 04, woman, age 60)

A participant also expressed that she became stronger through dealing with hostility:

“I was getting on a boat with my peers with stroke. The majority of us were walking with a stick … I heard someone say ‘These [disabled] people walk so slowly and are blocking my way. It's better for them not to go on trips.’ I was sad about it … but I told my friends that we hadn’t done anything wrong … This person just knew nothing and shouldn’t disturb our mood.” (Participant 11, woman, age 64)

These unpleasant experiences often left enduring impressions on survivors during recovery. Even afterwards, they continued to serve as reminders of their hard work, determination, and evidence of their past successes to overcome future challenges.

### 4.4 Theme 3: Engaging in life activities to better adapt to post-stroke challenges

Some participants revealed that they developed their individual rehabilitation methods at home, originating from ideas in daily life or participation in household, leisure, or social activities:

“I did worry about deterioration in health after quitting rehab classes to save money and travel, so I kept practising these exercises that I could remember at home, gradually I found these exercises were similar to some of my daily activities like walking sideways or cleaning tables … I knew I could continue my rehab at home.” (Participant 20, man, age 50)

“It is easy to lose self-function, isolate yourself and lose interest in everyday activities … persevere to go outdoors and do something that you would usually do before stroke, leisure or social activities, whatever you like, to keep you moving, and you will find what you need and the ways forward.” (Participant 09, man, age 54)

They further echoed the importance of self in the lifelong journey of post-stroke recovery. Regardless of support from others, stroke survivors needed to initiate and participate in the rehabilitative process. Stroke survivors had to make modifications, adjustments, or adaptations by themselves to suitably integrate rehabilitative exercises into their daily lives to address their specific health concerns. One participant elaborated that only she knew best the extent of her physical limitations and the appropriate ways to address these limitations to pursue targeted activities:

“After having a stroke, I was unable to draw pictures for a long time … My human drawings became asymmetrical as my vision deteriorated. I shortened my duration of drawing, drew more about scenes instead … my recent pictures look good. I can still enjoy drawing.” (Participant 06, woman, age 80)

Moreover, participants indicated that the key to successfully facilitating adaptation to post-stroke challenges was to keep exploring and evaluating the usefulness of strategies adopted. They consistently resynthesized general recommendations, modifying them in their own ways, and producing workable strategies that they felt comfortable to pursue regularly and integrate into individual lifestyles:

“You can find much information about training your arms, legs … but you cannot do them all, and also not all methods suit you. So, you need to screen and choose the way that suits yourself most.” (Participant 27, woman, age 69)

“The teacher taught me to pull my arm backwards to train my muscles, but I do not have a device like that in the gymnasium, then I asked my son to add this hanger at home, so I can continue the training.” (Participant 22, man, age 52)

To develop individually tailored strategies, participants also conveyed the need to continue to actively locate helpful resources. Resources should be easily located in daily life, including people, such as family members, friends, colleagues, healthcare professionals or care providers; tangible resources, such as financial support from community or government; rehabilitative services from health organizations or research studies; and news from newspapers, radios, or websites:

“I think this not only keeps me updated about the latest developments in healthcare, but also abreast of societal updates, finding my existence in the world.” (Participant 30, woman, age 59)

### 4.5 Theme 4: Being confident in oneself to persevere in self-management behaviors

Participants revealed that many stroke survivors often had low self-confidence post-stroke resulting from the trauma of the stroke, the sudden disruption to their life, and repeated failures in performing basic self-care activities because of physical limitations. Additionally, their confidence frequently fluctuated and was easily affected by different situations, for example, failures in performing self-care tasks, others’ wordings, the weather, and past memories. Consequently, confidence was a fundamental element in determining the sustainability of participants’ self-management behaviors:

“If you’re not feeling confident, you won’t be able to do anything, let alone to improve.” (Participant 04, woman, age 60)

Participants further suggested ways to build confidence. They stated that enhancing confidence required time and accumulation of successful experiences in performing particular health-related behaviors. To achieve this, they shared a common strategy of setting short-term personal goals:

“I’ll usually set a ‘small’ goal, say one week, for myself. For example, this week, I want to walk more steps steadily at home. If I can achieve this, I’ll be happy and more confident to do more. But if the goal is too big, I’ll give up easily as I have no hope of achieving it.” (Participant 01, man, age 35)

Participants indicated that confidence could not be strengthened without the encouragement of others, usually close significant others. Support may also come from others with similar conditions, who can better understand their unique shared experiences. Participants described the benefits of joining support groups for people with similar conditions, where they built confidence by learning from each other:

“I don’t mind sharing with others how I was after my stroke and how I trained myself hard, you see, I can walk with a stick now. I know the experiences and know very well how to encourage others with stroke to pass through the hard times.” (Participant 25, man, age 69)

However, some participants expressed concerns about attending group sessions, particularly at the initial stage post-stroke when they had limited physical ability, resulting in transport and safety concerns. They stated that their attendance was usually only possible based on the availability of others willing to accompany them or after a certain level of stability in mobility.

Although support from healthcare providers was regarded as important, participants mentioned not many opportunities to meet them after being discharged from the hospital, except for short meetings during follow-up consultations or routine physical training. They expressed that they would value more interactions, especially to receive expert health advice, enabling them to determine whether they were on the right track for rehabilitation:

“I like to join classes or talks run by health care providers. They are professional, and I trust their advice.” (Participant 30, woman, age 59)

Some participants relayed that supporting themselves was also very important when feeling defeated or losing confidence after negative experiences. They conveyed that the key was to change their way of thinking, reinterpret their negative experiences, and find positive aspects or learning points for further improvement. Participants indicated that it was more constructive to “appraise every tiny effort and accomplishment made for recovery” rather than to feel sad about not achieving their ultimate long-term goals. In this way, they could protect themselves from distressing voices and situations, gradually learning to feel satisfied with and enjoy all accomplishments.

Participants usually had vague and broad answers when asked about what they would like to attain. Some countered the question as they expressed fear of disappointment or over-focused on negative thinking about failures to attain their goals. Therefore, they felt that maintaining positive expectations about what they wished to attain was important, with 1 participant who liked to repeat:

“I hope to be able to achieve [some tasks] … to maintain this momentum in moving forward.” (Participant 10, man, age 52)

Survivors also emphasized cultivating a balance between outcome expectations and ability. They mentioned setting realistic goals and having a specific positive picture of what they wished to finally achieve helped push through hardships.

“How you think determines the level of achievement that you can attain.” (Participant 01, man, age 35)

### 4.6 Theme 5: Continuing to accept the current self and explore the new self

Participants commonly revealed that they felt lonely at home immediately post-stroke. Some even isolated themselves from friends or relatives by turning down visits and reducing participation in activities that involved anyone other than their immediate carers. However, they found that such isolation pushed them further into extreme emotional lows, and with time, they were not able to accept their current selves and others’ help and care. They felt that support and encouragement from family and friends helped encourage them to reintegrate into new or pre-stroke roles and to continue to pursue hobbies:

“The key to success is to accept it and think that this is life, and it is good that we are still alive. The only difference is that we may have to use different methods to attain what we need, try different ways from before and taste the differences in the process.” (Participant 11, woman, age 64)

“Find new ways of developing oneself, or find new roles, life and life circles, at least find something to enjoy.” (Participant 09, man, age 54)

Participants shared that they resumed or developed their post-stroke hobbies such as painting, calligraphy, pottery, singing, and knitting. They regarded these hobbies as a way to relieve themselves from the past loss, and more importantly, enabled them to establish their new self-identities by continuously exploring their potential abilities through enduring practice:

“My daughter encouraged me to do pottery. I think it's because of doing pottery, I was distracted and not very sad when I had my second stroke … People said I was great that I could use one arm to do pottery. I told them I trained myself to do so. No one said people with one arm can’t do anything.” (Participant 04, woman, age 60)

They also mentioned the importance of balancing accepting help from others and striving to do things on their own to maintain independence. Some stated that they needed to rely on their own life philosophy to move on with their life:

“I told myself and reminded myself to live in the moment, that you cannot get back to the past and cannot predict what will happen next. The best way is to concentrate on the present moment, this is the real thing you are dealing with.” (Participant 06, woman, age 80)

Participants, particularly those living with stroke for over 10 years, described their process of accepting their limitations and the irreversible consequences of the stroke. They felt this was critical before they could find new ways of pursuing life and reconstructing their new identity:

“Release the past and enjoy the present – accept who you are, and you will be able to learn from this moment to better prepare for the next.” (Participant 28, man, age 62)

Furthermore, participants felt that being curious was an effective way to distract themselves from their limitations. This drove them to focus on learning, gaining successful experiences, and building confidence. They also indicated that a “positive” aspect was that they had “extra” time available post-stroke, allowing them to process the details that they might have previously overlooked:

“Learn from the past to relieve concerns, worries, pain, regret, and find new meaning.” (Participant 12, woman, age 60)

Participants shared their experiences of helping others with similar conditions using their personal stories. Approximately half the participants were active in stroke support groups and/or other volunteer services for other stroke survivors. They regarded this as part of a new meaning or mission in their lives to contribute to society:

“I was helped by many people as I was recovering from stroke. I tell myself if anybody needs my help, I must help if I can.” (Participant 20, man, age 50)

“Helping others also reminds me to continue to work hard in learning and training myself … the help is mutual … I can serve as a role model to others in positively managing my stroke.” (Participant 01, man, age 35)

## 5 Discussion

This study shows that the journey after stroke is chronic, dynamic, and complex. Participants regarded learning as a prerequisite for improving their affected functions and managing uncertainties in recovery. Learning required self-participation, building self-efficacy and positive outcome expectations, testing and adapting strategies to one's own health conditions, being open to listening to and sharing experiences, celebrating one's own accomplishments, accepting the current self, and establishing a new self.

Our findings reaffirmed the importance of beginning and continuing self-management support for stroke recovery in hospitals and beyond. Participants widely acknowledged the importance of self in recovery and lifelong learning to adapt to post-stroke physical and psychosocial challenges. Consistent support was shown for the self-management concept, that those with chronic conditions manage their own health, revealing various self-supportive roles of stroke survivors such as learning and implementing new knowledge and skills to address their personal post-stroke challenges for attaining an optimal level of independence.^[20]^ Interestingly, however, the word “self-management” was seldomly mentioned in participants’ accounts, although most were implementing core self-management skills such as goal-setting, and problem-solving.^[12]^

Moreover, as iterated in previous studies, participants greatly valued confidence and self-efficacy to support hardships during recovery. A qualitative study of 10 stroke survivors, purposely selected from participants of a multicentered randomized controlled trial of outdoor mobility, found that successful reengagement in daily and social activities and attainment of previous and new meaningful roles were suggested to result in positive self-beliefs and confidence.^[21]^ Additionally, a systematic review of 17 articles reported that stroke survivors with higher levels of self-efficacy functioned better in daily activities, with self-efficacy more positively associated with mobility and balance, daily activities and health-related quality of life, and negatively associated with depression.^[22]^ Higher self-efficacy, particularly in psychosocial functioning, better prepares survivors to confront obstacles and adapt to their changed circumstances, leading to improved well-being.^[23]^ Strategies adopted by participants also echoed the sources of information of self-efficacy postulated by Bandura^[15]^: mastery of experiences through setting short-term goals, learning from others, verbal persuasion and reinterpretation of emotional or physiological arousals.

Importantly, participants were markedly supported by encouragement from others with similar conditions, consistent with a systematic review of 9 studies on the effectiveness of stroke self-management programs, which indicated that peer support could provide emotional assistance and validate the feelings and experiences of people living with chronic conditions.^[24]^ Our findings further highlighted that such group support experiences may be limited by survivors’ mobility, particularly at the initial stage post-stroke. Transportation problems and availability of companions were frequently mentioned as a reason for absence from community-based group sessions, as survivors experienced different degrees of physical disability, even after a mild stroke.^[25,26]^ One study developed videos of survivors sharing and conducted a randomized controlled trial with 128 adult stroke survivors, effectively enhancing their self-efficacy and outcome expectations as well as stroke self-management behaviors. The participants were encouraged to view the videos themselves during their free time to learn from others’ experiences.^[25,26]^ This strategy addressed the limitations of merely providing a one-off experience, literacy in reading written information, and transportation.^[27,28]^

While participants recounted the importance of setting goals, positive outcome expectations toward achieving the goal were concurrently emphasized. Outcome expectations indicated a clearer picture of achievement, serving as a drive to work harder, especially during times of wanting to give up, consistent with Bandura’ principles of outcome expectations.^[15]^ However, past research lacked evidence on the use of outcome expectations owing to greater focus on using self-efficacy principles in promoting health behaviors.^[29]^ Our findings, however, supported the use of both outcome expectations and self-efficacy. One randomized controlled trial of a new nurse-led self-management program reported that underpinning self-efficacy and outcome expectations into interventions to enhance stroke self-management was associated with improvements in self-management among 128 community-dwelling stroke survivors.^[26]^ Application of these principles in enhancing stroke recovery should be further explored.

We also found that some simple and pragmatic self-management strategies were available at home such as participating in everyday household activities, hobbies, and/or leisure activities. These strategies enabled survivors to better understand their limitations and abilities, and think of new ways to adapt to their needs. This finding supported those of previous studies, including a longitudinal qualitative study in the Netherlands that followed 10 stroke survivors for 2 years post-discharge and suggested that participants experienced and learned to self-manage their stroke consequences by exploring and adapting their performances and expectations via everyday activities in their own environments.^[30]^ In addition, survivors were encouraged to stay active at home, which aligned with the idea that at-home recovery is better.^[31]^ Because effective self-management requires ongoing decision-making on the most appropriate ways to address problems, continued strategy testing and modification also aligns with the self-management concept.^[12]^

Our findings showed that survivors gradually develop their own ways of living well with their physical limitations and increase their participation in self-management behaviors. They reconstructed their self-identity and found new meaning in their lives. A correlational descriptive study echoed that stroke survivors who had a decreased level of self-perceived burden had a higher level of participation in self-management behaviors.^[32]^

Survivors commonly find their new selves and continue to unleash their potential and contribute to society through volunteer service. Volunteer work provides a non-threatening environment for stroke survivors, and allows them to build confidence and reintegrate into the community.^[26]^ It also enhances social support, which was reported as a significant factor of reducing depression after stroke.^[33]^ Future studies could explore effective interventions using volunteer work to enable stroke survivors to help society and find meaning in life earlier in the recovery process.

## 6 Limitations

First, this study recruited a convenience sample of participants and the sample size was relatively small, thus limiting the generalizability of the findings to other survivors. Second, participants were recruited regardless of their recovery stage or stroke severity. Majority of them had stroke over 10 years. They have developed certain adaptive strategies to manage and hence live well with their stroke-related challenges. Their perceptions of impact of stroke may have been diluted over time. Hence, the findings were more on the positive side and do not identify specific adaptive strategies for specific stages, but instead provide an overview of the key elements and strategies used to facilitate recovery post-stroke. The met and unmet needs of survivors at different stages after a stroke including the period immediately after being discharged from the hospital should be investigated for a more comprehensive picture of coping strategies.

## 7 Conclusions

Stroke recovery is chronic and demanding. Survivors must learn new skills and knowledge to facilitate their participation in self-management behaviors. Providing self-management support and building self-efficacy and positive outcome expectations show promise for enhancing survivors’ abilities in self-management behaviors. The findings of this study may guide future research to develop more appropriate interventions for strengthening confidence and positive outcome expectations among stroke survivors. Innovative interventions to help survivors find meaning in life after stroke and better reintegrate into the society would also be valuable.

## Acknowledgments

The authors thank all the participants for sharing their experiences of recovery after stroke.

## Author contributions

**Conceptualization:** Suzanne Hoi Shan Lo, Janita Pak Chun Chau, Anne Marie Chang.

**Formal analysis:** Suzanne Hoi Shan Lo, Janita Pak Chun Chau.

**Funding acquisition:** Suzanne Hoi Shan Lo, Janita Pak Chun Chau, Anne Marie Chang.

**Investigation:** Suzanne Hoi Shan Lo, Janita Pak Chun Chau.

**Methodology:** Suzanne Hoi Shan Lo, Janita Pak Chun Chau, Anne Marie Chang.

**Project administration:** Suzanne Hoi Shan Lo.

**Resources:** Suzanne Hoi Shan Lo.

**Supervision:** Suzanne Hoi Shan Lo.

**Writing – original draft:** Suzanne Hoi Shan Lo, Janita Pak Chun Chau, Anne Marie Chang.

**Writing – review & editing:** Suzanne Hoi Shan Lo, Janita Pak Chun Chau, Anne Marie Chang.
